# A Singular Case of an Obstruction in the Ureters

**Published:** 1799-07

**Authors:** Charles Brown

**Affiliations:** Surgeon


### FOR THE MEDICAL AND PHYSICAL JOURNAL.

### *A ſingular Caſe of an Obſtruction in the Ureters.*

[Communicated by Mr. Charles Brown, Surgeon.]

JOHN WILSON, Eſq. a gentleman in the law, had laboured many years under ſevere fits of the ſtone, which at intervals were ſo violent, as to render him inſenſible. He had frequently paſſed ſeveral ſmall ſtones, with great pain. Laſt Monday evening, ſoon after I had viſited him, he was ſeized with cold ſhivering fits, a ſhooting pain in the bladder, extending up the rectum, attended with nauſea. Nothing was evacuated from the bladder, except now and then a ſpoonful of clear limpid liquor, which neither had the ſmell or taſte of urine. He was bled on the morning—took aperient and anodyne medicines during the day—and at night was put into the warm bath. The obſtruction continued till Tueſday, about four o'clock, P. M. when he became comatous, and died. Having obtained leave, I opened the body, when the following morbid appearances preſented to view, viz. the left kidney was conſiderably leſs than natural, and was become a thin ſac; the ureter belonging to it was very ſmall, and felt hard—when cut open, it was found filled with a gravel of a dirty colour, ſqueezed ſo cloſe together, that no liquor had probably paſſed that way for a conſiderable time. The right kidney was diſtended with urine to a monſtrous ſize, and the ureter was ſo large, that it had all the appearance of a portion of inteſtine. Upon opening it down to the bladder, I found a ſmall ſtone fixed ſo firmly between the coats of the bladder, that I had ſome difficulty to bring it out—it was not above a fourth of an inch from the orifice of the ureter into the bladder. The proſtrate gland was diminiſhed in ſize, and ulcerated; calculous concretions were formed in the urethra; theſe concretions were ſubmitted by myſelf to the following experiments:—

*Analyſis of the Concretions.*

A ſmall fragment being put into a drop of marine acid, on a piece of glaſs, over a candle, was ſoon diſſolved; and upon evaporation of the acid, cryſtallized in needles, making angles of about 60° and 120° with each other.

Water dropped on the cryſtals would diſſolve no part of them; but in marine acid they would re-diſſolve, and might be re-cryſtallized.

Vitriolic acid formed ſelenite with the calcareous earth.

By acid of nitrated quickſilver, phoſphoric acid was readily obtained.

When heated, theſe concretions decrepitate ſtrongly; they next emit the uſual ſmell of burnt animal ſubſtances, and were charned, but would not become white, though partially fuſed.

HATTON GARDEN, CHARLES BROWN.

*May 19th*, 1799.

